# Wideband Scattering Diffusion by using Diffraction of Periodic Surfaces and Optimized Unit Cell Geometries

**DOI:** 10.1038/srep25458

**Published:** 2016-05-16

**Authors:** Filippo Costa, Agostino Monorchio, Giuliano Manara

**Affiliations:** 1 Università di Pisa, Dipartimento di Ingegneria dell’Informazione, Pisa, Via Caruso 16, 56122, Italy

## Abstract

A methodology to obtain wideband scattering diffusion based on periodic artificial surfaces is presented. The proposed surfaces provide scattering towards multiple propagation directions across an extremely wide frequency band. They comprise unit cells with an optimized geometry and arranged in a periodic lattice characterized by a repetition period larger than one wavelength which induces the excitation of multiple Floquet harmonics. The geometry of the elementary unit cell is optimized in order to minimize the reflection coefficient of the fundamental Floquet harmonic over a wide frequency band. The optimization of FSS geometry is performed through a genetic algorithm in conjunction with periodic Method of Moments. The design method is verified through full-wave simulations and measurements. The proposed solution guarantees very good performance in terms of bandwidth-thickness ratio and removes the need of a high-resolution printing process.

The scattering control of objects is nowadays a strategic area of research. Most of the efforts in the last decade have been dedicated to the design of cloaking devices capable of guiding electromagnetic radiation around objects so as to making them invisible^1^,^2^,^3^,^4^. Cloaking technique allows also the minimization of scattering but its application is usually restricted to small objects and characterized by particular shapes^1^. The reduction of the electromagnetic signature of objects, in practice, is obtained by using a suitable geometry of the object^5^,^6^ or by employing electromagnetic absorbers^7^,^8^,^9^. The reduction of electromagnetic signature of objects represents also the main countermeasure against the most popular device based on retro-reflection, that is, the radar. Radar technology is presently applied to a wide range of scenarios including many military and civil applications. Some examples include aircraft radar, air traffic control, speed cameras, automotive high-resolution radar, radio frequency identification (RFID)^10^,^11^,^12^,^13^. While in a military scenario the main interest is to reduce Radar Cross Section (RCS) to make a target invisible, in other scenarios, such as RFID or self-driving cars^13^,^14^,^15^, the level of radar cross section may need to be increased to make obstacles more visible. The improved visibility of a target can be obtained through a scattering diffusion technique^16^,^17^,^18^,^19^,^20^,^21^,^22^,^23^. It consists in spreading of the energy towards multiple direction instead of a single one as it typically happens for planar surfaces. In general, in absence of losses, reflection cancellation towards the specular direction can be obtained by increasing cross-polar reflection or by increasing scattering towards non-specular directions.

One of the first approaches to achieve scattering diffusion was presented by Paquay *et al*. in 2007 who employed a combination of Artificial Magnetic Conductors (AMC) and Perfect Electrical Conductor (PEC) cells in a chessboard like configuration^16^. The approach has been then extended to wideband operation by using two AMC surfaces maintaining the opposition in phase within a certain band^17^. Aperiodic designs have been also proposed^18^.

Since energy spreading leads to a reduction of the bistatic contribution, these surfaces are often misleadingly proposed as an alternative to radar absorbing materials for reducing the visibility of a target. A typical interpretation for the radar cross section reduction is that the cheesboard surface induces scattering cancellation because of opposite phase values of closely located elements. However, it has to be remarked that cheesboard surfaces are designed by using an alternation of macro-cells formed by several elementary unit cells with a periodicity of macro-cells exceeding one wavelength. Alternative approaches consist in employing metamaterial coatings with randomly distributed refractive indices^19^. The latter has evident practical implementation problems due to the complexity. More recent papers have also proposed a design procedure exploiting the same theoretical background of reflectarray antennas^20^,^21^,^22^,^23^ but with a different purpose. Indeed, in this case the objective is not the energy focalization but the spreading towards generic directions. The scattering diffusion effect is obtained with artificial impedance surfaces with non-uniformly distributed reflection phases. The design approach has been proved to maintain reasonable wideband operation when used in conjunction with a stochastic optimization approach.

The present paper presents an innovative approach to obtain scattering diffusion. The approach relies on the use of an artificial impedance surface (AIS) comprising a frequency selective surfaces (FSS) placed in the vicinity of an impenetrable surface. The FSS is characterized by a periodicity exceeding one wavelength for inducing the excitation of a high number of Floquet harmonics. The scattering towards high order harmonics is maximized by optimizing the geometry of the unit cell through a genetic algorithm. The objective function used in the optimization process is the minimization of the reflection coefficient of the fundamental harmonic across a wide frequency band. In this way, the energy is efficiently spread towards high order harmonics in the whole analyzed frequency band. It is the first time, to the best of our knowledge, that periodic elements with large periodicities are employed to achieve wideband scattering diffusion. The proposed technique does not need subwavelength elements and therefore does not poses strict requirements in terms of precision of lithographic processes. This represents an advantage for mm-wave band and THz band but also, in general, when these surfaces are used in large objects with a size of tens of wavelengths.

The paper is organized as follows. Section II shows the theoretical background of the scattering from periodic impedance surfaces and the operating principle of the proposed scattering diffusion technique. In the third section, the description of the optimization process of the FSS geometry is addressed. Some representative results are reported in section IV. A visualization of scattered field through scattering patterns on wavevectors planes clarifies also the physical phenomena involved into the wave scattering of the proposed surface. Finally, section V reports measured results on manufactured samples compared to simulated results.

## Design approach a theoretical background

**Figure 1:**
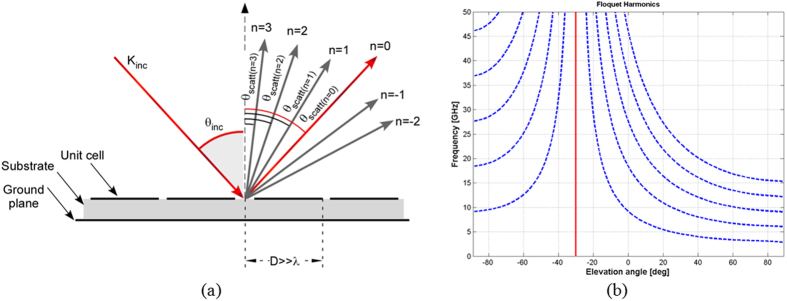
(**a**) Representation of scattering directions for an artificial impedance surface with repetition period exceeding one wavelength. (**b**) Scattering angle/frequency plot of scattered Floquet harmonics for an incident angle of θ = 30° and φ = 0°.

When an electromagnetic wave impinges on an interface it can be transmitted, reflected or absorbed. The direction of the reflected and transmitted waves follows the Snell law^24^. However, when the interface is a periodic surface with an inter-element distance exceeding one wavelength, interference phenomena lead scattered waves to be directed towards multiple directions known as diffraction orders or Floquet harmonics^7^,^25^,^26^,^27^. The propagation directions are solely determined by the geometry of the lattice on which unit cells of the periodic surface are disposed. In a one dimensional case, the non-specular directions of scattering can be computed according to basic theory of diffraction gratings^26^. With reference to Fig. 1a, due to the spatial periodicity, additional reflected components are characterized by the following in-plane wavevector components:
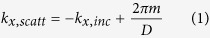


Dividing both members of (1) by the free space wavenumber *k*_*0*_, the scattering angles are derived:
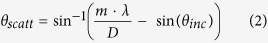
where *m* represents the harmonic number, *D* is the inter-element spacing, *λ* is the operating wavelength, and *θ*_*inc*_ is the angle of incidence. Let us suppose to have a periodic Frequency Selective Surface (FSS) printed at a certain distance from an impenetrable surface such that the transmitted energy is zero. This surface is known as Artificial Impedance Surface (AIS) and its geometry is reported in Fig. 1a. As an example, the direction of Floquet harmonics as a function of frequency, when θ_inc_ = 30°, is represented in Fig. 1b. Analogously, for a bidimensional (on *xy* plane) periodic array, the scattered field propagates towards the following directions:
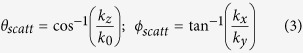
where *k*_*x*_, *k*_*y*_ and *k*_*z*_ are the *x*, *y* and *z* components of the wavevector *k*_*0*_ and are defined as follows^28^:
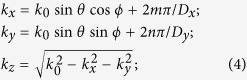
where *D*_*x*_ and *D*_*y*_ are the inter-element spacing along *x* and *y* directions, *(m, n)* represent the indexes of the Floquet harmonics, *θ* and *φ* represent the incident angle of the impinging wave within a spherical coordinate system. It is evident from the above relations that a certain number of higher order Floquet modes are in propagation when the FSS periodicity exceeds one wavelength. However, the degree of excitation of each Floquet harmonic depends on the specific shape of the FSS unit cell. The total scattered field can be reduced to a summation of harmonic contributions^26^:
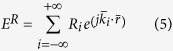
where *k*_*i*_ represents the wavevector of the *i*^*th*^ harmonic, *r* is the propagation direction and *R*_*i*_ represents the reflection coefficient of a specific harmonic, namely towards a specific direction. The energy associated to a specific harmonic is related to the diffraction efficiency^26^. Our objective is to maximize the diffraction efficiency of all excited high order harmonics over the largest possible frequency band and thus minimizing the scattered fields toward the bistatic direction. For simplicity, the scattering from impedance surfaces can be interpreted also by resorting to the theory of antenna arrays^20^. In this representation, the geometry of the element determines the element factor whereas the lattice geometry represents the array factor. If the scattering reduction has to be obtained in a narrow frequency band, the FSS geometry can, for instance, be designed by inspecting the surface current distribution but this method is not suitable for designing an FSS element characterized by wideband scattering reduction performance. The proposed strategy to obtain the desired wideband performance is to design the FSS geometry via an optimization algorithm in conjunction with a Method of Moment analysis of the impedance surface.

### Optimization process

As already pointed out in the previous section, the objective of this work is to design low-profile impedance surfaces able to spread the impinging energy via scattering diffusion and, as a consequence, drastically reduce bistatic reflection over a wide frequency band. To achieve this behaviour, a thin artificial impedance surface (AIS), whose stackup is reported in Fig. 1, is opportunely designed. The AIS surface is composed by a metallic backed substrate with a frequency selective surface printed on top. In order to excite a high number of Floquet harmonics and thus allowing multiple propagation directions, the periodicity of the FSS is imposed to be larger than one wavelength. In addition, the geometry of the FSS element is optimized to guarantee an efficient transferring of energy from fundamental Floquet harmonic to high order ones (high diffraction efficiency). The optimization of the FSS is performed though a genetic algorithm (GA)^29^,^30^. To this purpose, the FSS unit cell is discretized with a regular mesh composed by 16 × 16 pixels. The pixel can be made of metal or not and this binary information is stored in a 16 by 16 matrix. In order to focus the optimization process at the FSS geometry only, the dielectric substrate is *a priori* decided. The optimizer is supported by a Periodic Method of Moments (PMM) code based on roof-top basis functions^31^. The objective function consists in minimizing the reflection coefficient of the fundamental Floquet harmonic over a number of frequency points within the desired frequency band. The optimization process starts with an initial population of 1600 chromosomes which represent a set of possible solutions. Standard genetic operators such as selection, crossover, elitism and mutation are used to improve the solution, generation by generation^32^. The goodness of the solution is evaluated by using the following fitness function:
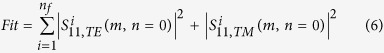
The number of frequency points *n*_*f*_ involved in the optimization process depends on the variability of the reflection profile with frequency. In the present case, we employed 21 points in order to follow the frequency variations adequately. The objective of the genetic algorithm is to find the FSS geometry which allows to minimise the fitness function (6). The algorithm converges to a stable solution after approximately 200 generations. However, it is not guaranteed that one run of the algorithm will provide the global best solution of the problem since there is the possibility that a local minimum is found^32^,^33^. The value of the fitness of the best solution is strongly related to the initial population. This is the reason why the optimization process is run several times and the global optimum solution is chosen after 100 runs of the genetic optimization process. A single run of the optimizer takes around five hours while the global optimization process can take several days. At the end of the optimization process, the unit cell geometry is double-checked to remove isolated pixels where any basis function is defined^29^.

Being the unit cell periodicity larger than the operating wavelength, issues about the accuracy of the mesh may rise. Indeed, the discretization of 1/16 with respect to the unit cell periodicity may not be sufficient if the periodicity is too large with respect to the operating wavelength. For this reason, the maximum allowed periodicity of 4λ at the maximum operation frequency is imposed and the accuracy of the optimal solution found by the GA algorithm is verified, at the end of the optimization process, with a 32 × 32 mesh. A direct optimization with a mesh of 32 × 32 is not feasible because of the prohibitive computation burden.

### Numerical Results

**Figure 2:**
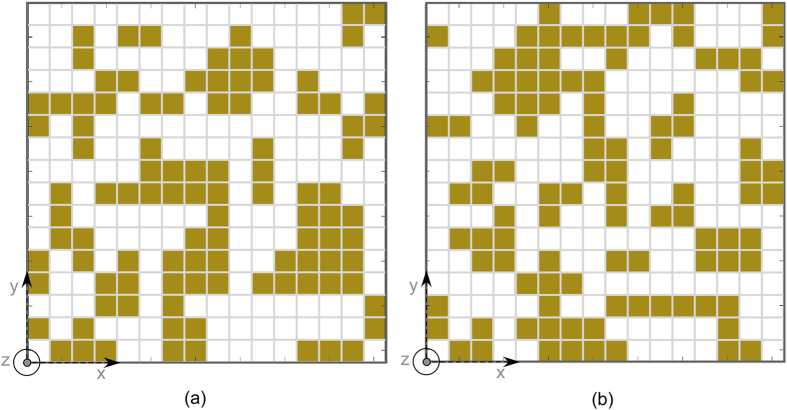
Two unit cell geometries obtained by optimizer. (**a**) Optimized geometry for with air substrate of 3 mm. (**b**) Optimized geometry for an FR4 substrate of 2 mm.

**Figure 3:**
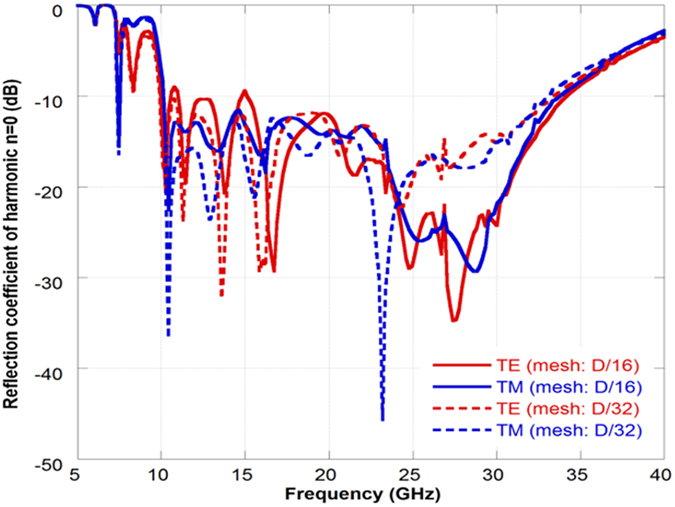
Reflection coefficient of the fundamental harmonic (m = 0, n = 0) for the air substrate configuration computed with a rough mesh (D/16) and with a more accurate one (D/32).

Two representative examples are shown to demonstrate the validity of the proposed approach. Both low and high permittivity substrates are employed to assess the effect of the substrate permittivity. In the first example, where an air substrate configuration is needed, the substrate is set to be a foam spacer (ε_r_ = 1.05) with a thickness of 3 mm. In this case, the periodic surface formed by the repetition of the optimized unit cell is etched on Kapton film. The thin layer is then glued on a Rohacell substrate backed by a metallic ground. The second design, namely the one employing a high permittivity substrate configuration, is formed by the optimized periodic surface printed on an FR4 substrate (ε_r_ = 4.4-j0.088) with 2 mm thickness and backed by a metallic ground. The optimized unit cell geometries are reported in Fig. 2. The unit cell geometries and the periodicity values are optimized through the genetic algorithm procedure described in the previous section. The periodicities are set to 40.5 mm and 29.2 mm respectively. The two different stackups are considered in order to understand the impact of the substrate permittivity on the bandwidth performance. The use of a low-permittivity substrate always provide better results in terms of bandwidth with respect to the high-permittivity substrate. For sake of brevity, the performance of the high permittivity configuration will be reported in the measurement section. The optimized reflection coefficient amplitude of the fundamental harmonic (m = 0, n = 0) for the air substrate configuration at normal incidence is reported in Fig. 3. The results obtained with the rough mesh used during the optimization process compares quite well with the more accurate solution obtained by using a considerable higher number of basis functions (discretization pixel equal to D/32).

A deeper analysis of the air substrate configuration in terms of RCS diagrams is necessary in order to understand the physical behavior of the designed device. To this purpose, a finite version of the designed impedance surface is analyzed by using Ansoft HFSS. The analyzed surface comprises a repetition of four by four unit cells. The geometry of the unit cell is the one shown in Fig. 1a. The repetition period is 40.5 mm and the total dimension of the surface is 162 mm^2^.

When the pixel based FSS, optimized through the genetic algorithm and simulated by the Periodic MoM approach, is simulated with another full-wave approach (e.g. Finite Element Method-FEM), the regions with a single point contact between adjacent pixels has to be adequately modelled. This aspect has been already treated in previous literature^34^,^35^. Since, there is no actual contact point in the MoM analysis (no basis function is defined between these pixels), a diagonal cut has been applied in the point of contact as suggested in^34^ (configuration b). The edge length of the small triangles subtracted from the square pixels are equal to one tenth of the pixel dimension, that is 0.25 mm in this case.

**Figure 4:**
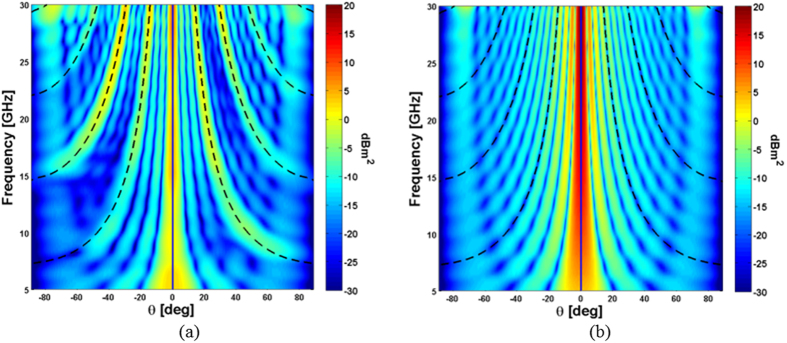
Radar cross section as a function of frequency and elevation incidence angle. The RCS is plotted on the plane φ = 0°. (**a**) Designed surface (air substrate configuration) and (**b**) PEC plane of the same dimension. The size of the surface is 162 mm^2^ and the impinging plane wave comes from θ = 0°, φ = 0°. Dashed lines represents the directions of high order Floquet harmonics according to relation (2).

In Fig. 4, the radar cross section of the artificial impedance surface is shown on a principal phi cut (φ = 0°) and as a function of elevation angle θ on the abscissa and as a function of frequency on the ordinate. The propagation direction of the Floquet harmonics, computed according to relation (2), are superimposed by using black dashed lines. As it is apparent, the designed surface provides an efficient spreading of the impinging energy towards the predicted high order harmonics contrarily to the metallic surface (Fig. 4b). The number of harmonics, as expected, increases proportionally to the operating frequency. Even if very useful, the phi-cut representation of the scattered fields does not predict all the possible propagation directions or, in other words, all the Floquet harmonics. Indeed, some of the energy is redirected towards directions outside of the principal planes. The additional harmonics can be easily predicted by using the relations (3) and (4) valid for the 2D case.

**Figure 5:**
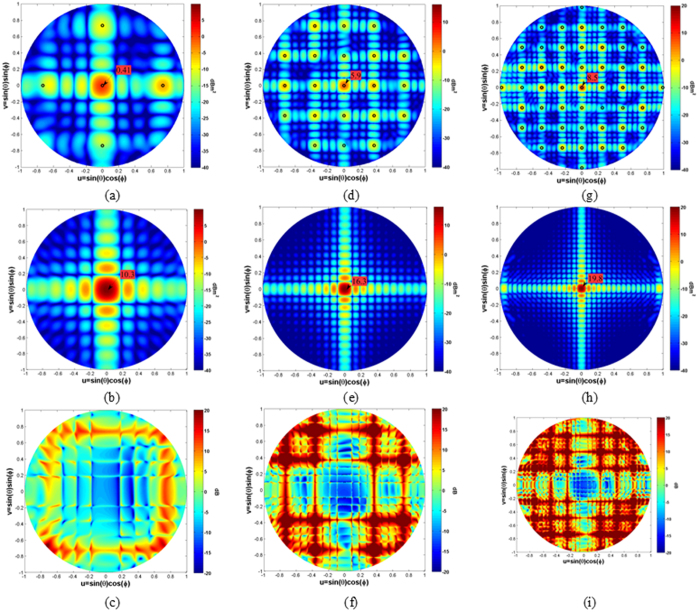
Scattering patterns on wave vector domain for the proposed artificial impedance surface, for a PEC surface of the same dimension (162 mm^2^) and difference between the two at three representative frequencies: (**a**) Metasurface 10 GHz, (**b**) PEC 10 GHz, (**c**) Difference 10 GHz, (**d**) Metasurface 20 GHz, (**e**) PEC 20 GHz, (**f**) Difference 20 GHz, (**g**) Metasurface 30 GHz, (**h**) PEC 30 GHz, (**i**) difference 30 GHz. The impinging plane wave comes from θ = 0°, φ = 0°. The peak value is reported within the figure.

To provide a clear visualization of the scattered energy over the phi and theta cuts, the scattering patterns on the normalized wavevector domain (u = sin(θ)cos(φ) and v = sin(θ)sin(φ)) are plotted in Fig. 5 for three representative frequencies (f = 10 GHz, f = 20 GHz, f = 30 GHz). The radar cross section of the designed surface is reported on the left whereas the radar cross section of a PEC surface with the same dimension is reported on the right for comparison. The incident angle of the impinging plane wave is set to θ = 0°, φ = 0°. The propagation directions predicted by the relations (3) and (4), superimposed on the colour plots by using black circles, agree very well with the one obtained with the numerical full-wave analysis. By simply subtracting the RCS of the impedance surface and the PEC surface, one can obtain the amount of reflectivity reduction/increasing. Indeed, as it is shown in Fig. 5 for the frequencies 10 GHz, 20 GHz and 30 GHz, the designed artificial impedance surface provides a reflectivity reduction of the central zone (bistatic reflection for normal incidence) and an increase of the scattered energy towards other angles.

**Figure 6:**
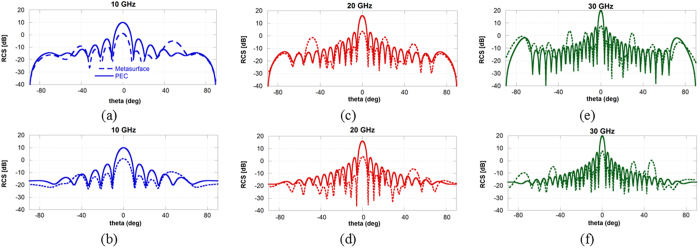
Scattering pattern of impedance surface and vs the PEC surface on the two main planes at three representative frequencies: (**a**) 10 GHz, φ = 0°, (**b**) 10 GHz, φ = 90°, (**c**) 20 GHz, φ = 0°, (**d**) 20 GHz, φ = 90°, (**e**) 30 GHz, φ = 0°, (**f**) 30 GHz, φ = 90°.

The scattered energy patterns become more uniform as the frequency increases because of the high number of excited harmonics. For an easier assessment of the difference between the scattered field of the proposed surface and the PEC plane, the conventional 2D Cartesian scattering patterns are shown in Fig. 6.

**Figure 7:**
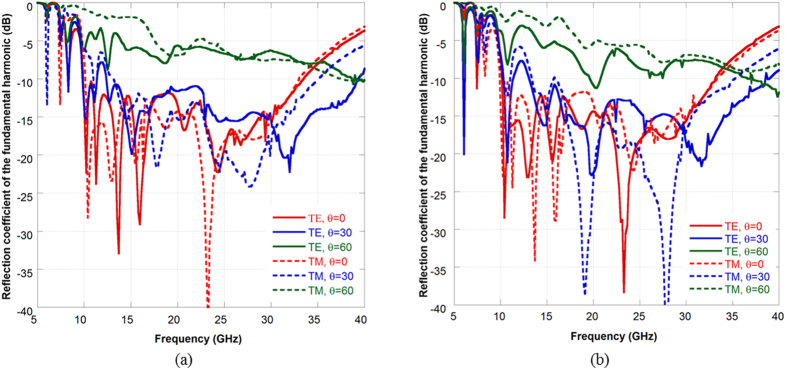
Reflection coefficient of the fundamental harmonic (m = 0, n = 0) for oblique incident angles. Impinging wave on cut (**a**) φ = 0° and (**b**) φ = 90°.

**Figure 8:**
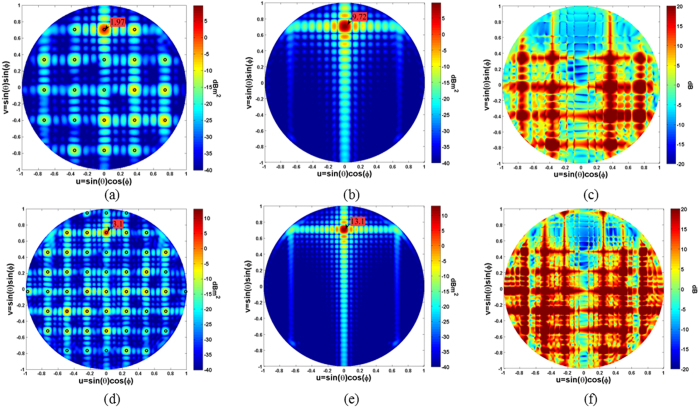
Scattering patterns on wave vector domain for oblique incidence. The impinging plane wave come from θ = −45°, φ = 90°. (**a**) Metasurface 20 GHz, (**b**) PEC 20 GHz, Difference 20 GHz, (**d**) Metasurface 30 GHz, (**e**) PEC 30 GHz, difference 30 GHz. The peak value is reported within the figure.

Even if designed for normal incidence, the surface maintains bistatic RCS reduction properties also for oblique incidence. In Fig. 7, the reflection coefficient of the fundamental harmonic (n = 0, m = 0) is shown for θ = 0°, 30° and 60°. The performance are reported in Fig. 6 both for a wave impinging on the phi-cut φ = 0° and on the phi-cut φ = 90°. The reflectivity reduction is stable up to 30° and then it degrades for θ = 60°. As previously shown, we can represents the scattering of the surface on the *(u, v)* domain to get a clearer visualization of the scattered fields. The plot are shown in Fig. 8 for two representative frequencies (20 GHz and 30 GHz) and for an impinging wave coming from θ = −45°, φ = 90°. The PEC plane redirects all the impinging energy towards the bistatic scattering angle predicted by the Snell law, whereas the impedance surface tends to spread the energy also towards additional directions determined by the propagating harmonics. The efficiency in spreading the energy towards the high-order harmonics depends on the shape of the unit cell. As before, the propagation directions predicted by using (3) and (4) are superimposed on the scattering pattern of the impedance surface by using black circles. The energy is indeed concentrated around the allowed propagation directions.

### Measured results

**Figure 9:**
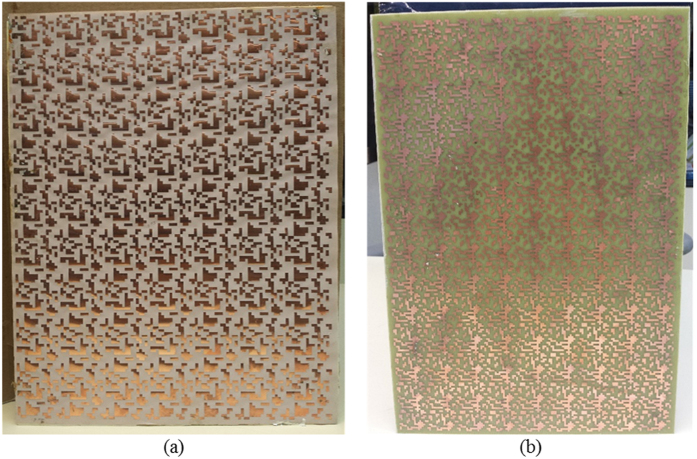
Pictures of the prototypes. (**a**) Air substrate configuration, (**b**) high permittivity substrate configuration.

To verify the reliability of periodic MoM simulations and FEM based simulations presented in previous sections, some prototypes have been manufactured and measured. The air substrate configuration, whose performance have been extensively discussed in the previous section, is manufactured by printing the FSS pattern on a thin Kapton substrate. The thin layer has been then glued to a 3 mm thick Rohacell substrate backed by an aluminum foil. The second prototype is made with a 2 mm thick FR4 and it has been fabricated to investigate the effect of using high-permittivity substrates. The photographs of the manufactured samples are shown in Fig. 9. The unit cell geometries are the ones shown in Fig. 1.

**Figure 10:**
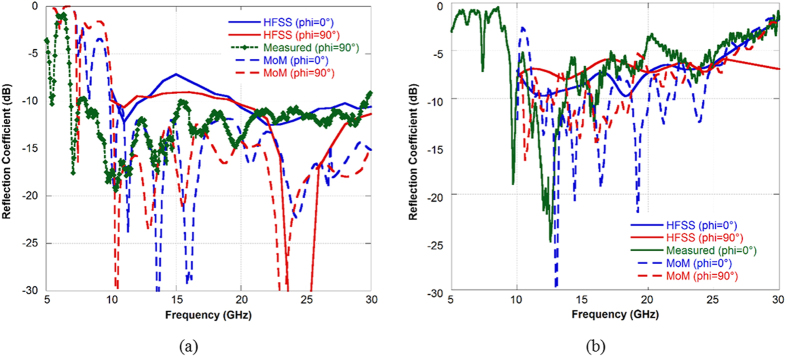
(**a**) Measured reflection coefficient at normal incidence for the finite impedance surfaces: (**a**) AIS with unit cell reported in Fig. 1a. The periodic surface is printed on a thin Kapton substrate and then glued to a grounded 3 mm Rohacell substrate backed by a metallic ground. The size of the manufactured sample is 285 mm by 366 mm. (**b**) AIS with unit cell reported in Fig. 1b. The periodic surface is printed on a 2 mm thick FR4 substrate backed by a ground plane. The size of the manufactured sample is 290 mm by 380 mm.

In Fig. 10 the measured reflectivity reduction of the impedance surfaces at normal incidence is shown. The results are compared both with the results of the MoM approach used for the optimization process and with the numerical results obtained by simulating the finite surface comprising 4 × 4 unit cells with HFSS. The results are in a reasonable agreement even if there are differences due to the infinite extent approximation and to the coarse mesh discretization used in the MoM approach. The use of a substrate with a higher permittivity allows reducing the thickness of the surface but it provides worse performance in terms of RCS reduction of the bistatic reflected fields.

## Conclusion

A novel method for obtaining diffusion of scattered fields towards generic directions is proposed. The approach exploits impedance surfaces comprised by frequency selective surfaces with a periodicity exceeding one wavelength. The geometry of the FSS cells is optimized by using a Genetic Algorithm in order to maximize the energy directed towards high order Floquet harmonics over a very-large frequency band. The scattering diffusion, characterized by a 10 dB reduction of the fundamental harmonic reflection, is obtained from 7 GHz to 30 GHz with a 3 mm thick artificial impedance surface. The scattering phenomenon is explained via Floquet theory and by using insightful colour scattering patterns on wavevectors domain. The results are confirmed by simulations and measurements on finite size samples.

## Methods

The response of the low-bistatic scattering surfaces have been measured through a couple of horn antennas operating between 2 GHz and 20 GHz. Two Teledyne Reynolds cables have been employed for connecting the antennas to the VNA. The cables introduce acceptable losses up to 26 GHz. The employed VNA is the PNA-L N5230C working up to 50 GHz. The measurements have been performed at normal incidence by using a bistatic configuration of the antennas. Even if the measured results can be considered reliable up to 20 GHz because of the equipment limitations (cables and antennas), they are plotted up to 30 GHz.

## Additional Information

**How to cite this article**: Costa, F. *et al*. Wideband Scattering Diffusion by using Diffraction of Periodic Surfaces and Optimized Unit Cell Geometries. *Sci. Rep.* **6**, 25458; doi: 10.1038/srep25458 (2016).
